# LiGenCam: Reconstruction of Color Camera Images from Multimodal LiDAR Data for Autonomous Driving

**DOI:** 10.3390/s25144295

**Published:** 2025-07-10

**Authors:** Minghao Xu, Yanlei Gu, Igor Goncharenko, Shunsuke Kamijo

**Affiliations:** 1 Graduate School of Interdisciplinary Information Studies, The University of Tokyo, Tokyo 113-0033, Japan; xu-minghao@g.ecc.u-tokyo.ac.jp; 2 Graduate School of Advanced Science and Engineering, Hiroshima University, Hiroshima 739-8527, Japan; 3 College of Information Science and Engineering, Ritsumeikan University, Osaka 567-8570, Japan; igor@fc.ritsumei.ac.jp; 4 Interfaculty Initiative in Information Studies, The University of Tokyo, Tokyo 113-0033, Japan; kamijo@iis.u-tokyo.ac.jp

**Keywords:** autonomous driving, generative adversarial networks, image reconstruction, LiDAR

## Abstract

**Highlights:**

**What are the main findings?**

- Color camera images can be realistically and semantically reconstructed from multimodal LiDAR data using a GAN-based model.
- The fusion of multiple LiDAR modalities enhances reconstruction quality, and the incorporation of a segmentation-based loss further improves the reconstruction fidelity.

**What is the implication of the main finding?**

- LiDAR can serve as a backup to cameras by reconstructing semantically meaningful visual information, enhancing system redundancy and safety in autonomous driving.
- LiGenCam has the potential to perform data augmentation by generating virtual camera viewpoints using panoramic LiDAR data.

**Abstract:**

The automotive industry is advancing toward fully automated driving, where perception systems rely on complementary sensors such as LiDAR and cameras to interpret the vehicle’s surroundings. For Level 4 and higher vehicles, redundancy is vital to prevent safety-critical failures. One way to achieve this is by using data from one sensor type to support another. While much research has focused on reconstructing LiDAR point cloud data using camera images, limited work has been conducted on the reverse process—reconstructing image data from LiDAR. This paper proposes a deep learning model, named LiDAR Generative Camera (LiGenCam), to fill this gap. The model reconstructs camera images by utilizing multimodal LiDAR data, including reflectance, ambient light, and range information. LiGenCam is developed based on the Generative Adversarial Network framework, incorporating pixel-wise loss and semantic segmentation loss to guide reconstruction, ensuring both pixel-level similarity and semantic coherence. Experiments on the DurLAR dataset demonstrate that multimodal LiDAR data enhances the realism and semantic consistency of reconstructed images, and adding segmentation loss further improves semantic consistency. Ablation studies confirm these findings.

## 1. Introduction

The automotive industry is now widely recognized for its software-intensive and safety-critical nature. The industry is progressing toward the ultimate goal of fully automated driving, beginning with relatively simple operational environments, such as highways. One of the most challenging, rapidly evolving, and crucial components of automated driving is the perception system, which enables vehicles to understand their surroundings and determine their own position relative to surrounding objects. Modern perception systems typically integrate multiple sensors and machine learning-based software. As automated driving systems (ADSs) transition from research to production, it is imperative to rigorously assess the safety of perception systems [1].

Highly automated vehicles must reliably maintain full control over safety-critical functions in various situations. Given that drivers may not always be fully attentive, these functions must be exceptionally dependable to avoid failures, particularly in Level 4 and higher autonomous vehicles (SAE Level 4 (J3016–2021) denotes an automated driving system that, within its defined Operational Design Domain (ODD), performs the entire Dynamic Driving Task and its fallback manoeuvres without human intervention; if a fault occurs or the vehicle is about to leave the ODD, the system must automatically bring the vehicle to a minimal-risk condition [2]). Experts recommend implementing redundant systems that incorporate fail-operational capabilities, ensuring that if one component fails, another can seamlessly assume control, allowing the vehicle to reach a safe state without human intervention. Such fail-operational designs are implemented in steering and braking systems, as well as in surround-perception sensor systems, control units, and electrical architectures [3].

The popular sensor configuration for perception in Level 4 and higher autonomous vehicles typically includes a combination of Light Detection and Ranging (LiDAR) sensors, radars, and cameras [4]. Each sensor serves a specific purpose: LiDAR and radar sensors measure distances and detect objects, while cameras handle visual identification tasks. Most existing research on perception systems assumes that sensors will not fail and that data from each sensor is collected accurately. However, these studies often do not adequately account for maintaining safety-critical functions in the event of sensor failures. The simplest approach to increasing system redundancy is through hardware, by installing duplicate sets of perception sensors. If one sensor fails, the other can assume its functions. However, this approach significantly increases the cost of the autonomous driving system and poses challenges in maintaining the vehicle’s aesthetic design due to the addition of extra sensors. An alternative software-based solution involves explicitly using data from one type of sensor to back up another. For example, camera image data can be used to reconstruct LiDAR point cloud data, which can then serve as input for perception algorithms, ensuring continuity in autonomous driving functions.

In recent years, significant research efforts have focused on reconstructing LiDAR 3D point cloud data from camera images. These studies typically utilize stereo [5] or monocular cameras [6,7] to estimate depth, subsequently reconstructing the LiDAR point cloud. Recent approaches have increasingly concentrated on enhancing the accuracy of depth estimation techniques by training deep learning models on extensive multimodal datasets containing synchronized images and LiDAR data [6]. However, research investigating the inverse process—reconstructing or translating LiDAR data into image data—remains relatively limited.

Therefore, this paper proposes a deep learning model named LiDAR Generative Camera (LiGenCam), which aims to effectively reconstruct image data from LiDAR data. The proposed model leverages Generative Adversarial Networks (GANs) [8] to reconstruct camera images from multimodal LiDAR data. Modern LiDAR sensors, particularly those employed in autonomous driving, robotics, and smart infrastructure, are capable of generating multimodal data that includes various perception modalities beyond traditional 3D point clouds [9]. The integration of ambient light sensing, reflectance measurements, and range imaging significantly enhances the perception capabilities of LiDAR systems. Unlike traditional methods relying primarily on a single modality, the proposed LiGenCam exploits this broader spectrum of multimodal LiDAR information to achieve improved reconstruction of camera images.

Maintaining semantic consistency between reconstructed camera images and their ground-truth is critical, especially as inaccuracies in essential elements such as vehicles and pedestrians can result in severe consequences in ADS. For instance, if the boundaries of the vehicle roadway or road markings are not accurately reconstructed, it may adversely affect drivable area detection and path planning, potentially leading to accidents in autonomous driving systems. To address this issue, we employ a pre-trained semantic segmentation model to compute semantic segmentation loss as the Kullback–Leibler (KL) divergence between the predicted segmentation distributions of reconstructed and ground-truth images. This approach ensures that the reconstructed images uphold not only visual fidelity but also semantic coherence.

To evaluate LiGenCam’s performance, this research employs three complementary metrics that assess both pixel-level and perceptual image quality: Peak Signal-to-Noise Ratio (PSNR), Structural Similarity Index Measure (SSIM), and Learned Perceptual Image Patch Similarity (LPIPS) [10]. Our experiments demonstrated a two-stage enhancement in reconstructed image quality. Initially, improvements were achieved by leveraging a broad spectrum of multimodal LiDAR data, including reflectance, ambient light, and range images, representing a significant advancement compared to traditional methods. Subsequently, incorporating the semantic segmentation-guided loss further optimized the PSNR, SSIM, and LPIPS scores, indicating an additional improvement in the perceptual quality of the reconstructed images.

The forthcoming sections of this paper will delve into the technical intricacies and practical implications of LiGenCam in greater detail. Section 2 comprehensively examines the related literature, setting the stage for our contributions. In Section 3, the specific model architectures of LiGenCam, encompassing both the generator and the discriminator, are delineated, along with a detailed discussion of the training objectives that guide our model. Section 4 presents the experimental framework, including dataset selection and preparation, followed by both quantitative and qualitative assessments of performance in Section 5. Section 6 explores the current limitations of our research and proposes avenues for future investigation. The paper concludes with Section 7, which synthesizes our principal findings and explores their broader implications.

## 2. Related Works

Reconstructing color camera images from LiDAR data for ADS is not an entirely new area of research. With objectives similar to ours, previous efforts in this field can be broadly divided into two categories: deep learning approaches that do not employ GANs (non-GAN-based) and those that incorporate GANs (GAN-based).

### 2.1. Non-GAN-Based Approaches

Moving into deep learning approaches that do not utilize GANs, an early attempt was presented in [11]. In that study, the authors employed an asymmetric encoder–decoder network to reconstruct camera images from LiDAR data. Their methodology primarily involved projecting LiDAR point clouds into reflectance images—2D projections of laser return intensity values that correlate with surface properties, as detailed in Section 4.2, and training a model to map these images to corresponding camera images.

An asymmetric structure was chosen to accommodate the disparity in data density between sparse LiDAR projections and denser camera images. Although demonstrating the potential of reconstructing camera images from LiDAR data, this approach encountered limitations due to its exclusive reliance on Mean Squared Error (MSE) as a similarity metric and its relatively simple architecture. Consequently, the method produced low-quality, blurry reconstructions, partly due to MSE’s tendency to average image details.

In a follow-up study by the same team [12], the authors enhanced their method by incorporating a selectively connected U-Net architecture [13]. This adaptation introduced skip connections exclusively near the network’s bottleneck, better aligning the information density between LiDAR point clouds and camera images. The U-Net architecture is particularly advantageous, as it preserves localization information, thereby improving the precision of the reconstructed images [14]. Although the refined approach still utilized MSE as the loss function, it produced higher-quality reconstructions with improved image clarity. Nevertheless, while these advancements addressed some shortcomings, fundamental limitations associated with relying solely on MSE and the relative simplicity of the architecture remained, highlighting the necessity for more sophisticated and nuanced models.

### 2.2. GAN-Based Approaches

The emergence of GANs, with their remarkable capabilities in image synthesis, has led to their application in domain translation tasks, such as translating LiDAR data to camera images in ADS.

The pioneering work in this field, as referenced in [15], utilized a conditional GAN [16] to convert upsampled point clouds into corresponding camera images. The upsampling was necessary to mitigate the sparsity of point clouds. However, the reliance solely on ranging information resulted in images of suboptimal quality.

A significant advancement was made in the study presented in [17]. This research introduced a conditional GAN framework incorporating three conditions: a point cloud scan of an object processed through PointNet [18] to extract features, a background patch, and the projected point cloud. They achieved considerably enhanced image quality by combining conditional GAN loss with L1 loss, a technique popularized by [19]. Nonetheless, the requirement for a clean scan of a single object restricts its applicability to scenarios akin to object placement and inpainting, making it less relevant for broader ADS objectives.

The work presented in [20] represents a more sophisticated and nuanced approach. This study integrated three existing networks [21,22,23] and introduced an additional network, forming a four-network framework. The primary aim was to train a model capable of translating LiDAR point cloud ranging information into semantic segmentation maps similar to those generated directly by camera-based segmentation models. Subsequently, the synthesized semantic segmentation maps were converted into camera images using Vid2Vid [23]. By explicitly incorporating semantic information, this method notably enhanced the preservation of critical scene objects, such as vehicles and pedestrians, which are often inadequately represented in existing reconstruction methods. However, a significant limitation arises from the method’s reliance on Vid2Vid for final image synthesis solely from semantic maps.

This is arguable because the method in [20] is designed for dataset augmentation and does not consider consistency with the real-world appearance of objects. Since semantic maps provide only pixel groupings according to semantic categories without preserving detailed textures, the images generated from semantic maps fail to maintain consistency with the real-world appearance of objects. Therefore, it is not an ideal option for on-road autonomous driving systems. This limitation remained unresolved in a follow-up study conducted by the same team [24].

### 2.3. Research Gaps

Few studies have focused on reconstructing camera images from LiDAR data, and among these, traditional non-GAN approaches often yield unsatisfactory results. Additionally, most GAN-based methods limit themselves to a single modality—typically either reflectance or ranging information from LiDAR sensor—lacking integration or comparison across multiple modalities. Moreover, only a single study, highlighted in [20], explicitly incorporates semantic information, which is crucial for maintaining semantic coherence in ADS, as discrepancies between reconstructed and actual images can lead to significant safety issues. However, this approach relies solely on limited semantic information provided by segmentation maps and thus fails to guarantee pixel-level similarity or preserve stylistic details in the reconstructed images. Recognizing these limitations, we propose LiGenCam, a conditional GAN-based model that leverages a broader spectrum of multimodal LiDAR data, including reflectance, ambient, and range modalities traditionally underutilized in this field. Our model integrates L1 loss with a semantic segmentation-guided loss to simultaneously ensure pixel-level accuracy and semantic coherence, bridging a critical gap left by previous methods that typically focus on only one of these aspects.

### 2.4. Relation to 3D Reconstruction Methods

Our work on 2D image reconstruction from LiDAR data shares the goal of generating photorealistic images with the field of 3D reconstruction and novel view synthesis, which is currently dominated by methods like Neural Radiance Fields (NeRF) [25] and 3D Gaussian Splatting (3DGS) [26]. However, a fundamental distinction lies in the methodology and primary objective. NeRF and 3DGS first build a dense 3D representation of the scene from a collection of input images with known camera poses. They then render new 2D views by querying this 3D model, enabling synthesis from arbitrary viewpoints.

In contrast, LiGenCam operates as a direct 2D-to-2D translation model. It does not construct an explicit intermediate 3D scene model. Instead, it learns a direct mapping from 2D projections of multimodal LiDAR data to a 2D camera image from a fixed, co-located viewpoint. The objective is, therefore, not novel view synthesis but rather sensor translation for redundancy—a critical failsafe application in autonomous driving. By forgoing the explicit 3D reconstruction step, our approach is more specifically tailored and potentially more efficient for this dedicated task, as the computational goal is to reconstruct a single, specific camera view rather than an entire 3D scene representation.

## 3. Approach

**Figure 1:**
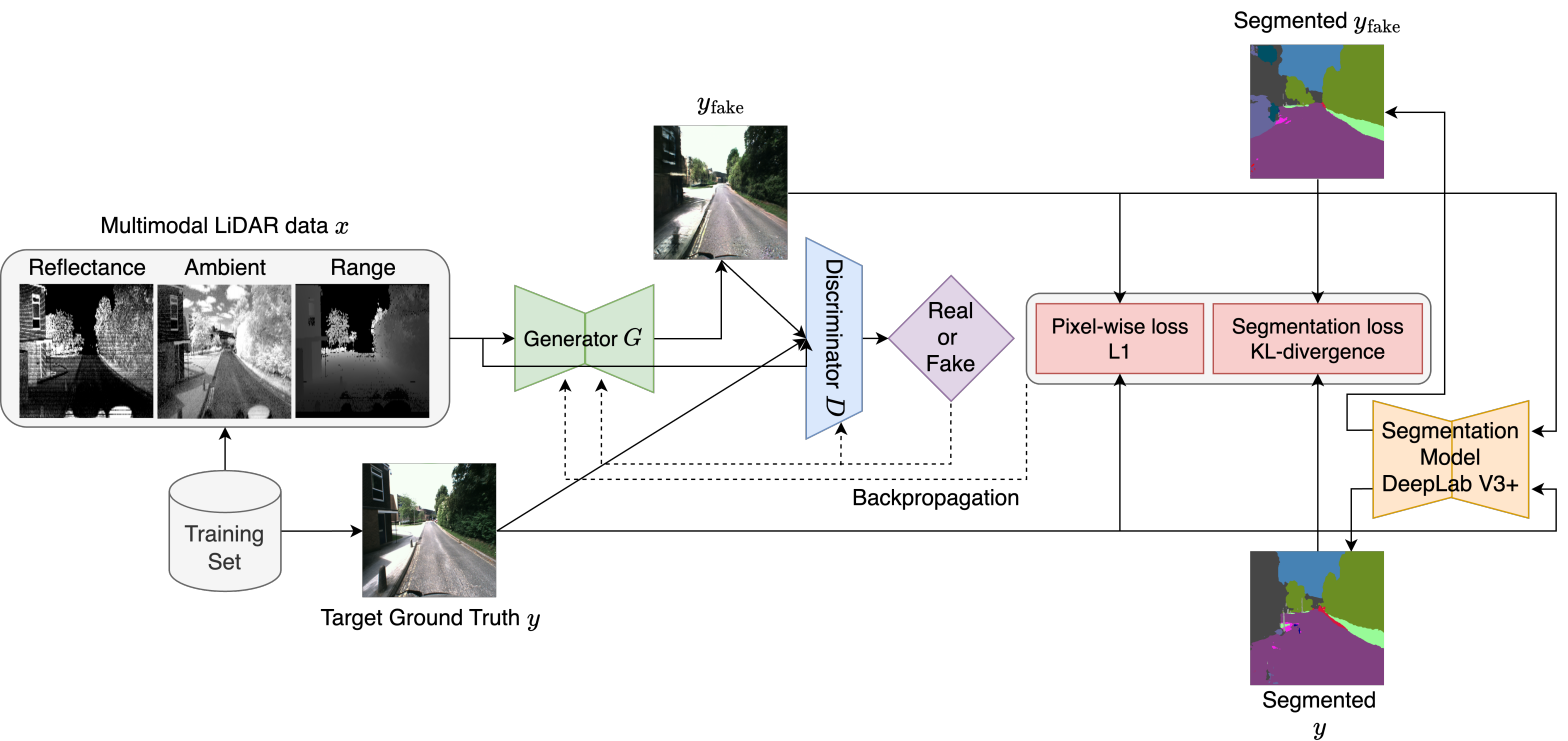
LiGenCam training phase. The model is a conditional GAN trained on paired data (x,y). The input *x* is a concatenation of three LiDAR modalities (reflectance, ambient, and range images), and *y* is the target ground-truth camera image. The generator *G* creates a fake image, $y_{\mathrm{fake}}$, from *x*. The discriminator *D* receives the input *x* as a condition to determine if an image (either the real *y* or the fake $y_{\mathrm{fake}}$) is a plausible match for that condition. To ensure semantic coherence, both *y* and $y_{\mathrm{fake}}$ are passed through a pre-trained segmentation model (DeepLabV3+) to compute a KL-divergence loss, which, along with a pixel-wise L1 loss, supervises the training.

LiGenCam, as depicted in Figure 1, operates on a fundamental principle: reconstructing camera images from multimodal LiDAR data while maintaining high fidelity to the original scene’s geometric and semantic attributes. This process begins with the input of two paired data points from the training set at the same timestamp—a target ground-truth camera image *y* and the corresponding multimodal LiDAR data *x*. In LiGenCam, *x* is a concatenation of multimodal LiDAR data: reflectance, ambient, and range image through the channel dimension, effectively capturing a comprehensive scene view.

While the reflectance and ambient modalities are direct panoramic image outputs from the sensor, the range image is generated by projecting the raw 3D LiDAR point cloud into a 2D representation, a process detailed in Section 4.3. The rationale for this particular data choice, concatenation style, and its impact on enhancing the model’s performance will be elucidated in Section 4.

The core objective is to synthesize a reconstructed camera image, denoted as $y_{\mathrm{fake}}$ (equivalent to G(x)), from *x*, using a modified conditional GAN. In this model, the generator produces $y_{\mathrm{fake}}$, which is then presented to the discriminator alongside the ground-truth image *y*. The discriminator’s task is to evaluate both images, discerning the reconstructed from the real. Feedback from the discriminator is essential for optimizing both the generator and the discriminator. Initially, both may perform suboptimally, but with iterative training and refinement, the generator is expected to evolve in its ability to produce images that closely match real camera images. Once the discriminator can no longer differentiate between real and synthesized images, the generator is considered sufficiently trained and ready for inference on unseen data.

To ensure geometric consistency, a pixel-wise L1 loss is computed between $y_{\mathrm{fake}}$ and *y*. For semantic coherence, both $y_{\mathrm{fake}}$ and *y* are passed through a pretrained segmentation network DeepLabV3+ with MobileNet backbone [27], and the segmentation results are compared via an additional segmentation KL-divergence loss. These additional loss functions are instrumental in further optimizing the generator and discriminator.

Throughout this paper, we consider paired samples (x,y)∼p(x,y), where *x* is the multimodal LiDAR data and *y* is the target camera image. In certain formulas, only *x* appears explicitly, in which case we may write x∼p(x) to represent the marginal distribution. However, all samples ultimately come from (x,y)∼p(x,y).

Detailed explanations of model architectures, as well as thorough descriptions of the model objectives, will be provided in Section 3.1 and Section 3.2.

### 3.1. Model Architectures

Our choice of a GAN framework over simpler regression models, such as a U-Net trained solely with a pixel-wise L1 or L2 loss, is motivated by established findings in the image-to-image translation literature [19]. It is well-documented that relying exclusively on pixel-wise losses tends to produce blurry results by averaging all plausible outputs. The adversarial loss component in a conditional GAN, however, learns a loss function that penalizes unrealistic images, thereby encouraging the generation of sharper and more perceptually convincing details.

LiGenCam leverages GANs, which have revolutionized generative models in Computer Vision (CV), as evidenced by significant works such as [28,29,30]. GANs have demonstrated superior generative capabilities compared to traditional encoder–decoder networks. GANs are particularly well-suited to LiGenCam’s objectives. This alignment is due to their ability to effectively synthesize high-quality images, making them an ideal choice for the complex task of reconstructing camera images from multimodal LiDAR data. A typical GAN is fundamentally composed of two neural networks: a generator *G* and a discriminator *D*, trained in an adversarial fashion. Our model builds upon the conditional GAN (cGAN) framework. To provide context, we will first outline the principles of a standard GAN before explaining how the introduction of a conditioning variable leads to the cGAN architecture that our model adapts.

In GANs, *G*’s role is to learn the distribution of the training data and synthesis samples that are indistinguishable from real data, starting from a random noise vector *z*. This noise can be considered a seed from which the *G* learns to synthesize new data. In contrast, *D* aims to accurately distinguish between real data and the fake samples generated by *G*. The objective is to refine *G* to the extent that it manufactures samples that *D* identifies as real or fake with a probability of 50%. This signifies that *D* no longer differentiates between real and generated samples, marking an optimal point where *G*’s outputs are convincingly realistic. The original GAN framework utilizes Binary Cross Entropy (BCE) loss, which quantifies how far the *G* is from its goal of producing realistic data and how well the *D* is performing in identifying real and fake data. The training dynamics are usually referred to as a min–max game, mathematically represented as:

(1) $$\min_{G}\max_{D}V\left(D,G\right)=E_{y∼p(y)}\left[\log D(y)\right]+E_{z∼p(z)}\left[\log\left(1-D(G(z))\right)\right].$$

where *y* denotes a sample from the real data distribution, *z* is the input noise for *G*, usually is sampled from a Gaussian distribution, and V(D,G) is the value function for this min–max game.

While the standard GAN framework is powerful, it does not inherently offer control over the characteristics of the generated samples. Conditional GANs address this limitation by introducing a conditioning variable *c* into the training process. This variable, which can be a one-hot label, an encoded text prompt, or any informative attribute, guides the generation process, allowing for more targeted and controlled output. Such targeted control over the generative process is usually what is desired, as it enhances the model’s applicability and effectiveness in real-world applications.

In conditional GANs, the generator function becomes G(c,z), incorporating both the random noise *z* and the conditioning variable *c*. The discriminator, in turn, evaluates not only the authenticity of the generated samples but also their alignment with the condition *c*. The min–max game for conditional GANs reflects this added complexity:

(2) $$\min_{G}\max_{D}V\left(D,G\right)=E_{\left(x,c)∼p(x,c\right)}\left[\log D(x|c)\right]+E_{z∼p(z),\;c∼p(c)}\left[\log\left(1-D(G(z)|c)\right)\right].$$

This equation indicates that *D* assesses the likelihood of a sample being real given the condition *c*, while *G* aims to produce samples that are not only realistic but also meet the specified condition.

In LiGenCam, we aim to reconstruct camera images in a deterministic manner, contrasting with the usual stochastic sampling of conditional GANs. Each multimodal LiDAR input *x* corresponds to a unique target camera image *y*, motivating a strict one-to-one mapping. Consequently, we omit the random noise *z*—which is typically used for stochastic generation—and focus on G(x) as the direct translation from LiDAR data to camera images. The formulation for our adapted GAN is as follows:

(3) $$\min_{G}\max_{D}V\left(D,G\right)=E_{\left(x,y)∼p(x,y\right)}\left[\log D(y|x)\right]+E_{x∼p(x)}\left[\log\left(1-D(G(x)|x)\right)\right].$$

In this formulation, G(x) represents the reconstructed camera image derived from the input multimodal LiDAR data *x*, while *y* represents the corresponding target camera image, serving as the training reference for *D*.

The loss functions for the generator and discriminator in our adapted model are detailed in the following subsections.

#### 3.1.1. Generator Loss Function

The generator loss function is given by:

(4) $$L_{\mathrm{GAN}}=-E_{x∼p(x)}\left[\log D\left(G(x)|x\right)\right].$$

This loss function maximizes the likelihood that *D* classifies G(x) as real. It encourages *G* to produce camera images from multimodal LiDAR data indistinguishable from actual camera images, thus fine-tuning *G* to closely replicate the real data.

#### 3.1.2. Discriminator Loss Function

The discriminator loss function is given by:

(5) $$L_{D}=-E_{\left(x,y)∼p(x,y\right)}\left[\log D\left(y|x\right)+\log\left(1-D\left(G(x)|x\right)\right)\right].$$

This loss function serves as a dual objective: to accurately classify both the real images *y* and the generated images G(x). The first term, logD(y|x), assesses *D*’s ability to recognize real camera images, while the second term, log(1−D(G(x)|x)), evaluates its proficiency in identifying images generated by *G* as fake.

In our implementation, the total loss for the discriminator $L_{D}$ is calculated by taking the mean of the loss from real images and the loss from fake images, expressed as:

(6) $$L_{D}=-\frac{1}{2}\;E_{\left(x,y)∼p(x,y\right)}\left[\log D\left(y|x\right)+\log\left(1-D\left(G(x)|x\right)\right)\right].$$

This method of averaging the losses effectively regulates the learning rate of the discriminator, ensuring that it does not become overwhelmingly dominant over the generator. Such calibration is to address the prevalent issue of mode collapse in GANs. Mode collapse occurs when the generator discovers shortcuts to deceive the discriminator, often by producing a limited variety of outputs [31]. While these repetitive outputs might successfully trick the discriminator, they represent an impractical convergence for real-world applications.

The above adaptation allows LiGenCam to produce camera images that are not only contextually relevant to the given input conditions but are also deterministic in nature.

#### 3.1.3. U-Net as Generator

U-Net [14], initially developed for biomedical image segmentation, also gained popularity in the domain of GANs as a generator for image-to-image translation tasks. Its architectural edge lies in its ability to effectively preserve critical structural and localization information, compared to traditional encoder–decoder networks where such details are often lost, particularly at the bottleneck stage. U-Net’s innovative use of skip connections circumvents this issue by directly linking the encoding layers to the decoding layers, ensuring that essential details are not only retained but also seamlessly integrated into the final output. This capability is crucial for high-fidelity image generation applications, where maintaining accuracy and detail is paramount.

In LiGenCam, U-Net’s [14] architecture is well-suited for translating multimodal LiDAR data into high-resolution camera images. Its effectiveness stems from its capability to handle situations where the input and output, while different in appearance, share a common underlying structure. This characteristic is central to LiGenCam, where the input multimodal LiDAR data and the output target camera images are different representations of the same physical environment.

**Figure 2:**
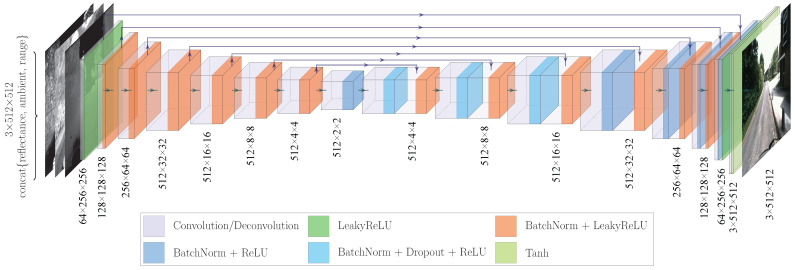
Architecture of the 16-layer U-Net in LiGenCam.

As depicted in Figure 2, our U-Net is designed to process tensors of shape 3×512×512, suitable for both the multimodal LiDAR data (in actual implementation, a concatenation of reflectance, ambient, and range image) as input and the target camera images as output. The choice of this specific tensor shape strikes a balance between computational efficiency and the network capacity to capture sufficient detail. Here, the dimension 512×512 represents the spatial resolution, and 3 is the number of channels. For the input tensor, each of the three channels corresponds to a distinct modality of the multimodal LiDAR data, effectively encoding different aspects of the original scene. In contrast, for the output tensor, the three channels represent the standard RGB (red, green, blue) color of camera images. This specific delineation of channels in both the input and output tensors underscores that, fundamentally, our network’s generator is engaged in an image-to-image translation task.

We adopt a 16-layer U-Net architecture. This design choice is grounded in established practices for image-to-image translation tasks, drawing inspiration from seminal works like Pix2Pix [19]. A 16-layer depth, consisting of eight downsampling and eight upsampling blocks, provides a sufficiently large receptive field to capture global contextual information in the scene, which is crucial for realistic reconstruction. At the same time, it maintains a manageable model size, ensuring that training remains computationally feasible on our hardware (an NVIDIA RTX 2080 Ti with 12 GB of VRAM) without prohibitively long training times or excessive memory demands. This represents a pragmatic trade-off between model capacity and practical resource constraints.

The network comprises two principal pathways: the downsampling path and the upsampling path, each constructed from key units known as downsampling and upsampling blocks, respectively. The downsampling blocks, roughly characterized by a series of convolutions, batch normalization, and LeakyReLU activation, form the downsampling path. In line with conventional practices in network design, where a convolution layer is followed by batch normalization, we set the bias parameter of the convolution layers to false. This is because the batch normalization process effectively neutralizes the need for the bias term in the convolution layers. This downsampling path excels in extracting intricate features from the multimodal LiDAR data. Conversely, the upsampling path is assembled from upsampling blocks, which roughly consist of deconvolution (transpose convolution), batch normalization, and ReLU activation. We also use a few dropout layers. These blocks focus on reconstructing the extracted features back into high-resolution camera images. This structured configuration ensures that the multimodal LiDAR data undergoes a coherent and accurate transformation into detailed camera images in alignment with the U-Net’s intended functional architecture.

Integral to our U-Net’s functionality are the skip connections, which link each layer in the downsampling path to its corresponding layer in the upsampling path. They bridge each layer in the downsampling path with its corresponding layer in the upsampling path, concatenating the activations from layer i with those from layer n−i (for a total of n=16 layers). These connections are instrumental in preserving essential low-level details and expediting the learning process by promoting a more efficient flow of information across the network.

The network’s output layer comprises a deconvolution and a Tanh activation function. The Tanh function normalizes pixel values to a range [−1,1], ensuring the output images maintain key visual qualities like texture and contrast.

#### 3.1.4. PatchGAN as Discriminator

A discriminator’s traditional role involves evaluating the output data entirety, usually producing a single scalar value indicating if the data is “real (1)” or “fake (0)”. While effective for general purposes, this approach can sometimes overlook the finer details and subtle nuances present in high-resolution images.

LiGenCam implements a variation, PatchGAN [32], to this process as its discriminator. Instead of producing a single scalar, PatchGAN is a fully convolutional network that outputs a 2D grid of scores. Each score in this final grid assesses the realism of a large, overlapping patch of the input image. The name “N×N PatchGAN” refers to the size of this receptive field, not the dimensions of the output grid. In our specific architecture, the discriminator processes the input image through a series of convolution layers to produce this grid of scores.

The patch size, *N*, is a significant hyperparameter because it dictates the scale at which the model assesses authenticity. A small *N* would force the discriminator to focus only on fine-grained, local textures, potentially missing larger structural inconsistencies. Conversely, a very large *N* (approaching the full image size) would behave like a traditional discriminator, evaluating global coherence at the expense of local detail. The choice of N=70, following the original and widely adopted implementation of PatchGAN, represents an effective trade-off: it is large enough to capture meaningful local structures (such as windows, road markings, or parts of a vehicle) and penalize common GAN artifacts, yet small enough to encourage the generation of sharp, high-frequency details throughout the image.

**Figure 3:**
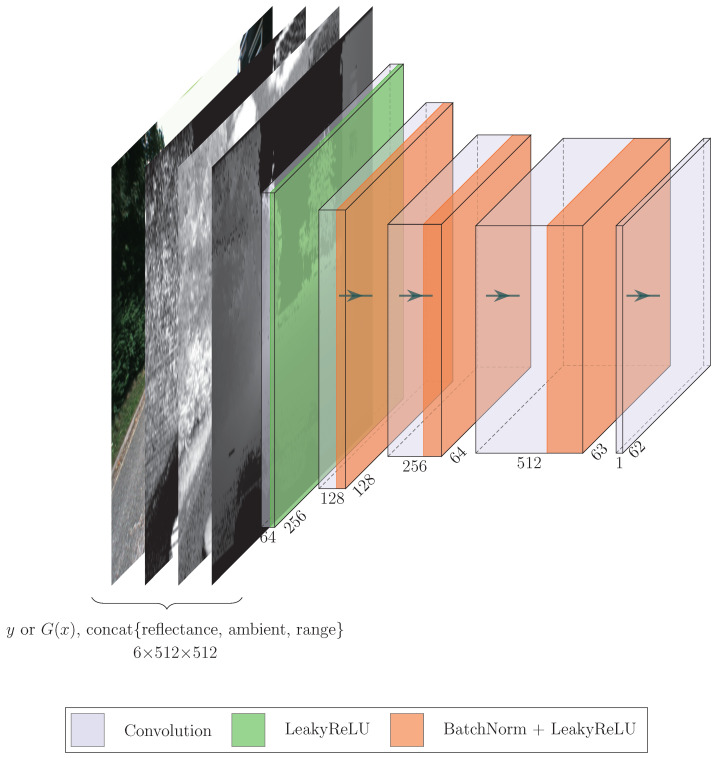
Architecture of the 70×70 PatchGAN in LiGenCam.

In our implementation, as depicted in Figure 3, we utilize a 70×70 PatchGAN. This approach entails that each element in the resulting 62×62 output grid corresponds to a 70×70 patch in the original input image. For our specific application, the input tensor has a shape of 6×512×512, and the output matrix has a shape of 1×62×62. This configuration is due to the input being a concatenation of reflectance, ambient, and range image (three channels) from the LiDAR data, along with the target camera image (three channels), as the discriminator requires both the condition (multimodal LiDAR data) and the target for effective discrimination. The architecture of our PatchGAN is composed of a sequence of convolution blocks. Each block integrates a convolution layer, batch normalization, and LeakyReLU activation. The final convolution layer in this series is tasked with producing the 62×62 output grid.

### 3.2. Model Objectives

The primary goal of LiGenCam is to reconstruct camera images that closely resemble the ground-truth, while simultaneously maintaining their geometric and semantic attributes. To achieve this objective, we define the model’s learning targets through the integration of three distinct loss terms: modified conditional GAN loss (BCE), pixel-wise loss (L1), and segmentation loss (KL-divergence). The modified conditional GAN loss, which ensures the generation of realistic images, has been previously detailed in Section 3.1.1. In the following sections, we focus primarily on the functions of the additional L1 and KL-divergence losses, which are instrumental in preserving the geometric similarity and semantic coherence of the reconstructed images.

#### 3.2.1. L1 Loss

We align with a common practice in GAN training by integrating a more traditional loss, specifically the L1 loss, alongside the GAN objective, a combination found effective in studies such as [33], further exemplified by [19]. The L1 loss, or least absolute deviations (LADs), is crucial for quantifying absolute differences between the reconstructed and target images at the pixel level, thereby maintaining geometric similarity. The rationale behind this practice is that using only the GAN loss can lead to excessively sharp images while relying solely on L1 loss often results in blurred outputs [19]. Thus, the combination of both L1 and GAN losses offers an optimal balance, enhancing image sharpness and detail while preserving overall realism.

The mathematical formulation of the L1 loss we use is as follows:

(7) $$L_{L1}=E_{\left(x,y)∼p(x,y\right)}\left[|y-G(x)|\right].$$

Here, G(x) denotes the reconstructed image from the generator given multimodal LiDAR data *x*, *y* is the target camera image. In our model design, we chose L1 loss over L2 because it treats all errors uniformly, unlike L2 loss, which emphasizes larger errors and often leads to overly smooth outputs. This uniform treatment of errors means that L1 loss does not disproportionately penalize larger discrepancies between the predicted and target images. As a result, it better preserves the edges and fine details in the generated images, leading to outputs that are sharper and more detailed.

By integrating L1 loss, LiGenCam not only ensures the generation of images that are visually realistic but also achieves pixel-wise similarity.

#### 3.2.2. KL-Divergence Loss

Integrating L1 loss effectively ensures pixel-wise similarity, providing more visually accurate images. However, in the context of ADS, it is imperative to not only focus on this aspect but also to prioritize semantic consistency. The accurate representation of vital elements like pedestrians and vehicles is of utmost importance, as any misrepresentation could have severe consequences.

Standard generative models often struggle with achieving this level of semantic accuracy. The primary challenge is that understanding and interpreting the semantics of a scene goes well beyond the capabilities of simple pixel-wise comparisons, typically quantified by metrics such as L1 or L2 norms (like we did previously at Section 3.2.1). While useful for assessing general image similarity, these conventional metrics fall short of capturing the essence of critical objects. Therefore, an image with a low L1 norm compared to its target might still inaccurately represent essential components, which is a significant shortfall in actual ADS applications.

Semantic segmentation maps are widely adopted solutions to ensure semantic consistency in image-to-image synthesis. This approach generates segmentation maps for synthesized and real target images using a pre-trained segmentation model. The segmentation maps obtained categorize pixels into a set of predefined semantic classes. These maps are then utilized to calculate discrepancies, forming the basis of a loss function that guides model optimization through backpropagation. Essentially, this process steers the generative model to produce images that, upon segmentation, yield maps that closely resemble those derived from the original images.

**Figure 4:**
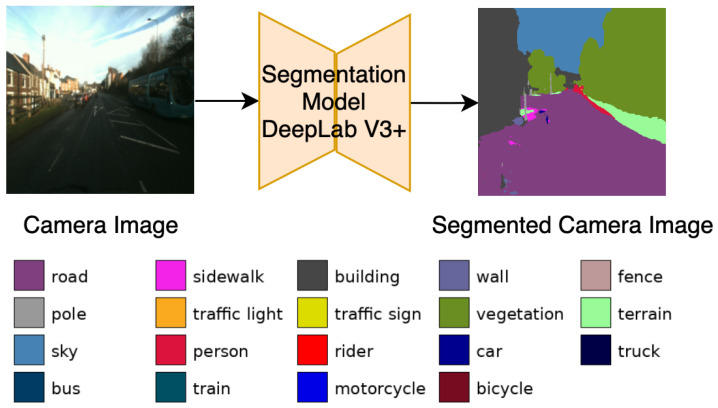
Example showing how semantic segmentation fosters semantic fidelity in LiGenCam. Each of the 19 Cityscapes classes is color-coded, with a corresponding legend for clarity.

As illustrated in Figure 4, our pre-trained DeepLabV3+ on Cityscapes (MobileNet backbone) partitions images into 19 semantic categories (e.g., road, sidewalk, person, car), each highlighted by a distinct color. In LiGenCam, to preserve the semantics of critical objects (e.g., pedestrians, vehicles), both the synthesized image G(x) and the corresponding target image *y* are passed through this segmentation network. At each pixel (u,v), the network produces a probability distribution over the 19 classes, denoted as $p_{\mathrm{synth}}\left(k|G\left(x_{u,v}\right)\right)$ and $p_{\mathrm{real}}\left(k|y_{u,v}\right)$, where k∈{1,…,19} indexes the Cityscapes semantic classes. To penalize deviations in these distributions, we adopt the Kullback–Leibler (KL) divergence:

(8) $$L_{\mathrm{KL}}=E_{\left(x,y)∼p(x,y\right)}\left[\sum_{u,v}\sum_{k}p_{\mathrm{real}}\left(k|y_{u,v}\right)\log\frac{p_{\mathrm{real}}\left(k|y_{u,v}\right)}{p_{\mathrm{synth}}\left(k|G(x_{u,v})\right)}\right].$$

By minimizing this divergence, we encourage the generator to produce outputs whose segmentation distributions match those of the real images, thereby securing both the appearance and semantic coherence of key objects. Consequently, LiGenCam not only yields images that are visually realistic (through L1 and GAN losses) but also largely maintains critical semantic details essential for ADS applications.

#### 3.2.3. Final Objectives

Throughout previous sections, we have elaborated on the integration of a modified conditional GAN loss (BCE), L1 loss, and KL-divergence loss into LiGenCam. This integration introduces two new hyperparameters: $\lambda_{L1}$ and $\lambda_{\mathrm{KL}}$. The $\lambda_{L1}$ parameter controls the extent to which pixel-wise accuracy is enforced, while $\lambda_{\mathrm{KL}}$ dictates the degree of semantic consistency enforcement. Adjusting these parameters allows for nuanced tuning of the model’s focus on maintaining semantic accuracy, which is pivotal for preserving essential elements of the scene.

Consequently, the final objective function for our generator can be formulated as a weighted sum of these three loss components. The generator aims to minimize the following composite loss function:

(9) $$L_{G}=L_{\mathrm{GAN}}+\lambda_{L1}\;L_{L1}+\lambda_{\mathrm{KL}}\;L_{\mathrm{KL}}.$$

where the individual loss terms are defined in the original Equations (4), (7) and (8).

## 4. Experiments

### 4.1. Selection of the Multimodal Dataset DurLAR

In this research, the DurLAR dataset [34] was selected for several compelling reasons. The primary distinguishing feature is its vertical resolution of 128 LiDAR beams, notably surpassing the resolution of commonly used datasets in the autonomous driving domain. For instance, the KITTI dataset provides LiDAR with 64 vertical beams [35], while the nuScenes dataset, another frequent choice in the field, offers a lower resolution of just 32 beams [36]. The enhanced resolution provided by the DurLAR dataset is particularly beneficial for our image reconstruction tasks. It allows for a more detailed and nuanced 3D representation of the environment, thereby improving the accuracy of the reconstructed images.

The selection of the DurLAR dataset was driven by our core research question: whether the quality of reconstructed images can be improved by fusing multimodal LiDAR data—specifically, reflectance, ambient, and range images. While other widely used datasets, such as KITTI and nuScenes, provide point clouds with reflectance values, they lack the panoramic ambient light image stream, which is essential for our study. Since our central hypothesis posits that the synergy among all three modalities (reflectance, ambient, and range) enhances reconstruction performance, the DurLAR dataset was uniquely suited to address this research question. Notably, some leading LiDAR manufacturers already offer sensors capable of outputting reflectance, ambient, and range data simultaneously, further underscoring the practical relevance of our chosen modality fusion.

**Figure 5:**
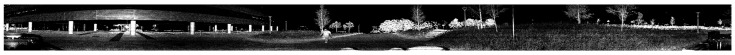
Panoramic reflectance image from the DurLAR dataset, captured at the same timestamp as Figure 8. The image resolution is 1×2048×128, featuring a single channel with 16-bit depth.

**Figure 6:**
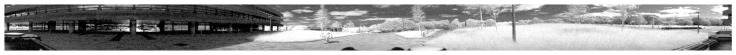
Panoramic ambient image from the DurLAR dataset, captured at the same timestamp as Figure 8. The image resolution is 1×2048×128, featuring a single channel with 16-bit depth.

**Figure 7:**
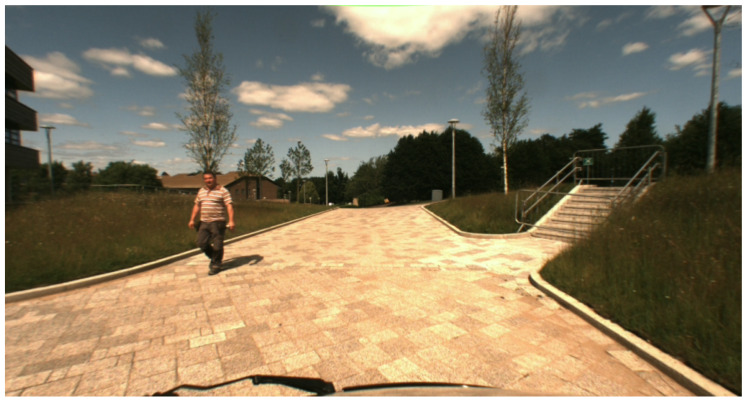
Color camera image from the DurLAR dataset, captured at the same timestamp as Figure 8. The image resolution is 3×1024×544, featuring three channels with 8-bit depth.

The dataset is comprehensive, encompassing a variety of modalities, including the following:

- Point Clouds: Offering 3D spatial information about the surroundings, this modality captures the environment’s structure with high precision. The point cloud format is KITTI-compatible, in (x,y,z,r), where *x*, *y*, and *z* represent coordinates and *r* denotes reflectance strength.
- Reflectance Panoramic Image: This modality is crucial for understanding the interaction of light with various materials in a scene. It provides detailed information about the reflectance properties of objects. (see Figure 5).
- Ambient Panoramic Image: Operating in the near-infrared spectrum, this modality adeptly captures environmental nuances under diverse lighting conditions, excelling particularly in low-light scenarios. (see Figure 6).
- Camera Image: Captured in the visible spectrum, this modality offers a conventional, human-eye-like perspective of the environment. (see Figure 7).
- GNSS/INS: While this data provides precise geo-referencing and motion tracking, its primary utility is in navigation and positioning tasks. It is less directly applicable to our focus on image reconstruction.

**Figure 8:**
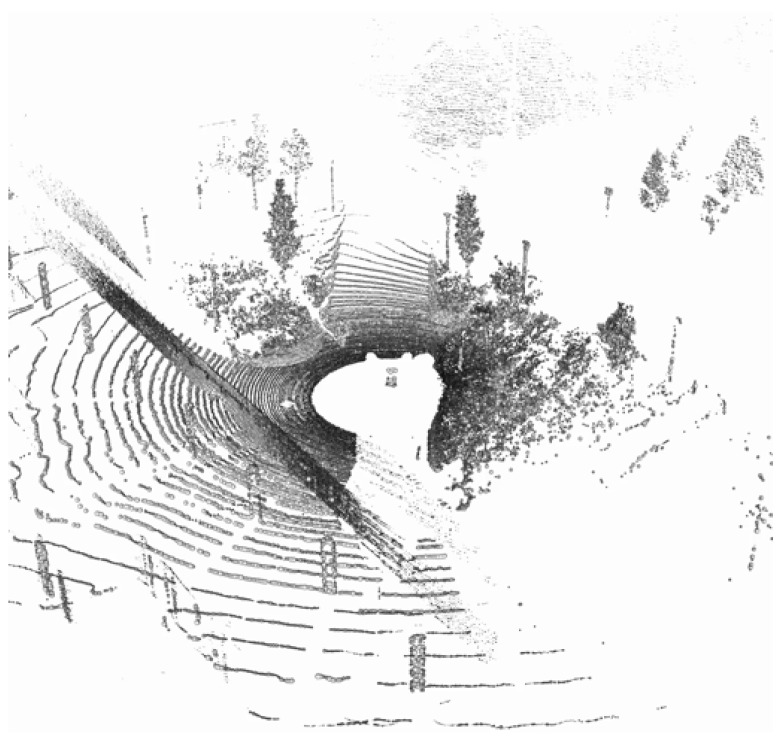
Rendered LiDAR point clouds from the DurLAR dataset.

The DurLAR dataset also boasts a notable advantage in its wide diversity of environmental and weather conditions, even though it follows a consistent driving route. Training models with a broad spectrum of conditions cultivates robust generalization, vital for effectiveness in various real-world scenarios.

Furthermore, the considerable size of the DurLAR dataset represents a significant benefit. Comprising 145,911 data points across different modalities, its substantial volume makes it particularly suitable for our image reconstruction task.

### 4.2. Selection of Modalities and Fusion Techniques

Multimodal data fusion is a common and effective strategy for enhancing model performance in ADS. As extensively reviewed in [37], current approaches to multimodal fusion can be primarily categorized into two types: strong fusion and weak fusion. Strong fusion techniques, encompassing methods like early, deep, and late fusion, involve the direct integration of various modalities at different stages of the processing pipeline. This approach aims to leverage the complementary strengths of each modality to enrich the model’s understanding and interpretation of data. On the other hand, weak fusion utilizes data from one modality as a supplementary signal to refine or guide the processing of another modality. While multimodal fusion holds great potential for boosting model performance, it also introduces challenges, such as managing the heterogeneity of different data types and addressing the increased computational demands. These challenges necessitate careful consideration in selecting and applying fusion techniques to ensure optimal performance and efficiency.

In selecting data modalities for our study, we incorporated raw point clouds, reflectance panoramic images, ambient panoramic images, and camera images from the DurLAR dataset. We decided to exclude GNSS/INS data, as the location and motion information provided offers minimal benefit for our image reconstruction task. Additionally, we refrained from developing an end-to-end model for direct LiDAR-to-image translation due to computational limitations. Instead, LiDAR point clouds are projected into range images, a common modality providing essential distance information regarding objects such as vehicles and pedestrians in relation to the LiDAR sensor.

Ambient image (near-infrared) is crucial for capturing global illumination variations, essential in diverse lighting conditions, especially in low-light environments. Reflectance images play a critical role in our image reconstruction task, particularly for colorization, since none of the chosen input modalities contain color information. Although reflectance does not capture color in the RGB sense, it helps differentiate surfaces and materials, thereby guiding the generator in estimating plausible color distributions. It uncovers material textures, improving the model’s ability to interpret and reconstruct complex scenes with enhanced detail. Camera images are utilized as the target ground-truth. Each modality has its unique strengths and limitations. Our approach, which diverges from traditional methods primarily focused on reflectance, incorporates three distinct modalities. This integration enriches our model with a broader spectrum of information, enhancing its capacity to reconstruct images and scenes more accurately and effectively.

For the fusion of the chosen modalities, we have employed strong early fusion techniques. This combines reflectance, ambient, and range images at an early stage through channel concatenation, ensuring that the modalities are integrated before being introduced to the model for processing.

The subsequent sections will explore the methodologies used for data preparation, including generating range images from LiDAR point clouds and for the effective concatenation of the selected three modalities.

### 4.3. Data Preparation from the DurLAR Dataset

The DurLAR dataset provides the primary data modalities for our work. We utilize the rectified camera images from the dataset, which is essential for ensuring a precise geometric correspondence between the projected LiDAR data and the ground-truth imagery. The dataset also provides panoramic reflectance and ambient images. The generation of range images from raw LiDAR point clouds involves several mathematical transformations. The steps involved in this process are detailed below:

**Step 1: Radial Distance Calculation**

The radial distance *r* for each point P(x,y,z) in the point cloud is computed using the Euclidean distance formula:

(10) $$r=\sqrt{x^{2}+y^{2}+z^{2}}.$$

**Step 2: Azimuth and Elevation Angle Calculation**

The azimuth angle θ and elevation angle ϕ for each point are derived from their Cartesian coordinates:

(11) $$\begin{array}{cc} \theta & =atan2(y,\;x)+\alpha , \end{array}$$

(12) $$\begin{array}{cc} \phi & =atan2\left(z,\;\sqrt{x^{2}+y^{2}}\right). \end{array}$$

Here, α represents a predefined angle offset, set to align the point cloud with the camera’s perspective. α is manually calibrated to −4 degrees, which is converted into radians for use in the calculations.

**Step 3: Field of View Filtering**

Points outside the LiDAR sensor’s vertical FoV are excluded. The FoV is specified by ${\mathrm{FoV}}_{\mathrm{upper}}$ and ${\mathrm{FoV}}_{\mathrm{lower}}$, representing the upper and lower angular boundaries of the sensor’s vertical range. Only points where ϕ falls within these boundaries are used in the image formation process. The ${\mathrm{FoV}}_{\mathrm{upper}}$ is set at 22.5 degrees and the ${\mathrm{FoV}}_{\mathrm{lower}}$ at −22.5 degrees, both of which are converted into radians in the calculations.

**Step 4: Image Coordinate Mapping**

The azimuth and elevation angles, θ and ϕ, are mapped to pixel coordinates in the range image. In this mapping, the horizontal axis (x-coordinate) in the image correlates with the azimuth angle, while the vertical axis (y-coordinate) corresponds to the elevation angle. The image width and height are denoted as *w* and *h*, respectively. The resulting pixel coordinates, $x_{\mathrm{img}}$ and $y_{\mathrm{img}}$, are calculated as follows:

(13) $$\begin{array}{cc} x_{\mathrm{img}} & =\left(w-1-\frac{\theta +\pi }{2\pi }\;w\right)\;\mathrm{mod}\;w, \end{array}$$

(14) $$\begin{array}{cc} y_{\mathrm{img}} & =\left(h-1-\frac{\phi -{\mathrm{FoV}}_{\mathrm{lower}}}{{\mathrm{FoV}}_{\mathrm{upper}}-{\mathrm{FoV}}_{\mathrm{lower}}}\;h\right). \end{array}$$

**Step 5: Radial Distance Mapping to Image Intensity**

The radial distances are scaled and mapped to image intensities to create the final panoramic range image:

(15) $$\mathrm{range}\left[y_{\mathrm{img}},x_{\mathrm{img}}\right]=\frac{r}{D_{\max}}\;I_{\max}.$$

**Figure 9:**
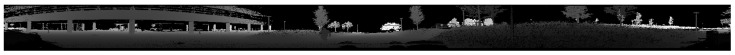
Projected panoramic range image from the DurLAR dataset raw point clouds.

Here, $D_{\max}$ represents the sensor’s maximum observable distance, set to 40 m, which corresponds to the 99th percentile of the observable range. $I_{\max}$ is $2^{16}$, which denotes the highest intensity value in a 16-bit image format. This 16-bit format is consistent with the ambient and reflectance images in the dataset. Figure 9 is an example of the projected panoramic range image.

**Step 6: Cropping, Resizing, and Concatenation:**

The data from the reflectance, ambient, and processed range modalities undergo cropping, resizing, and concatenation along the channel axis. This adjustment is necessary due to the differing image sizes of the modalities: the camera images have a resolution of 3×1024×544, while the other modalities initially have a resolution of 1×2048×128. The cropping ensures that the FoV of the reflectance, ambient, and range images aligns with that of the camera images. In practice, we horizontally crop the LiDAR-based images from X=750 to X=1330 pixels to match the camera’s FoV. The dimensions of these modalities are slightly adjusted to be larger than those of the camera images, ensuring comprehensive coverage. The resizing is performed to standardize the image sizes across all selected modalities and be compatible with the input requirements of the U-Net detailed in Section 3.1.3. (The actual 512×512 resize is applied later in our training pipeline on the fly. The script in Step 6 handles only the horizontal cropping.)

This process results in a comprehensive, multimodal input for LiGenCam, combining the distinct features of each modality into a single, unified input:

(16) x=[Reflectance,Ambient,Range].

**Figure 10:**
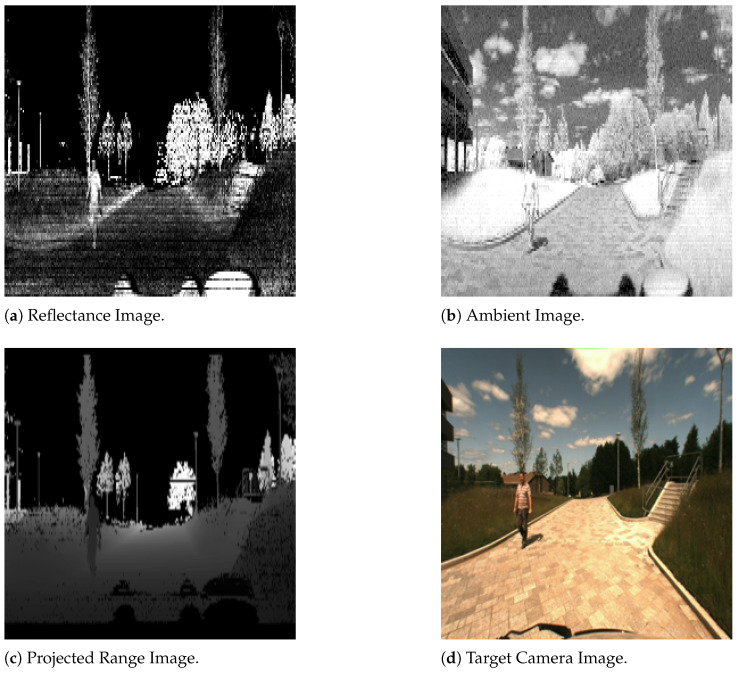
Processed multimodal LiDAR data and the corresponding target camera image. (**a**) The reflectance image captures surface properties. (**b**) The ambient image provides near-infrared intensity. (**c**) The projected range image encodes distance information. (**d**) The target RGB camera image serves as the ground-truth.

The final shape of the concatenated and resized modalities is thus 3×512×512, standardizing the input format through early fusion. Figure 10a–c correspond to channels 1, 2, and 3 of a single input sample, respectively. In summary, LiGenCam learns to map from this multimodal input to the target camera image in Figure 10d.

### 4.4. Training Details

We train LiGenCam for 50 epochs on the DurLAR dataset, which contains 145,911 frames collected across five different dates. To create our training and test sets, we employ a custom random temporal splitting procedure. This method randomly selects frames from the complete timeline to serve as test candidates. Crucially, to prevent data leakage and ensure a robust evaluation, a “buffer zone” is created around each selected test frame: the test frame itself and its ±5 temporal neighbors are excluded from both the final training and test sets. This strategy ensures the model is not evaluated on frames that are nearly identical to its training data. The selection is random and does not explicitly stratify by location or environment; we rely on this randomized sampling over the large dataset to obtain a representative distribution. In practice, this process results in approximately 3–5% of the original frames being allocated to the test set, with about 30% being discarded in the buffer zones. We then crop and resize each remaining sample to a final resolution of 512×512, resulting in roughly ∼95k training samples.

Both the generator (U-Net) and the discriminator (PatchGAN) employ the Adam optimizer [38] with a common GAN setup ($\beta_{1}=0.5$, $\beta_{2}=0.999$, initial learning rate $2\times 10^{-4}$) and a cosine annealing schedule to gradually reduce the learning rate. Each mini-batch contains 18 samples, and we accumulate gradients over 2 iterations (effective batch size of 36). All model training, testing, and specific computational efficiency benchmarks (the FPS measurement) were conducted on an NVIDIA RTX 2080 Ti with 12 GB of VRAM. A typical training run required about two days.

We normalize the input data to a floating-point range of [0, 1] based on its bit depth. The 16-bit LiDAR inputs (reflectance, ambient, and range) are divided by $2^{16}-1$, while the 8-bit camera images are divided by $2^{8}-1$. Automatic mixed precision [39] further reduces GPU memory usage and speeds up training. As in our original setup, the loss function combines a conditional GAN objective with a weighted pixel-wise L1 term ($\lambda_{L1}=20$) and a segmentation-based KL-divergence term $\lambda_{\mathrm{KL}}=2\times 10^{-5}$ using a DeepLabV3+ backbone. The segmentation loss is applied only after epoch 10 to avoid early instability. Following a coarse random hyperparameter search (learning rates, epoch scheduling, loss weights, etc.), LiGenCam consistently converges to high-quality reconstructions capturing both the geometry and semantics of real driving scenes. We track training and validation losses in real time with Weights & Biases [40].

## 5. Results and Discussion

In this section, we present a comprehensive evaluation of LiGenCam’s performance, based on the experimental framework detailed in Section 4. Our assessment is twofold: we first conduct a quantitative analysis using established image quality metrics to objectively measure reconstruction fidelity. This is followed by a qualitative assessment, where we visually inspect and compare the generated images to highlight the practical implications of our findings.

### 5.1. Quantitative Evaluation

A key challenge in evaluating image-to-image translation models arises from the paired nature of the task. Unlike unconstrained generative modeling, where Fréchet Inception Distance (FID) is often used to compare distributions of real and synthesized images [41,42], one-to-one frameworks benefit more from direct comparisons with their corresponding ground-truth targets. Consequently, we adopt three complementary metrics that capture both pixel-level and perceptual quality: PSNR, SSIM, and LPIPS.

PSNR and SSIM quantify pixel-level fidelity and structural similarity, respectively, and are widely adopted in image restoration and compression tasks. Higher PSNR implies closer intensity matching, while higher SSIM indicates stronger structural preservation. LPIPS, in contrast, measures perceptual similarity in a learned feature space and tends to align more closely with human subjective quality. Lower LPIPS, therefore, implies images that appear closer to the target visually. In practical ADS contexts, no single metric fully reflects semantic correctness and aesthetic quality, so balancing these three helps provide a more robust measure of reconstruction fidelity.

**Figure 11:**
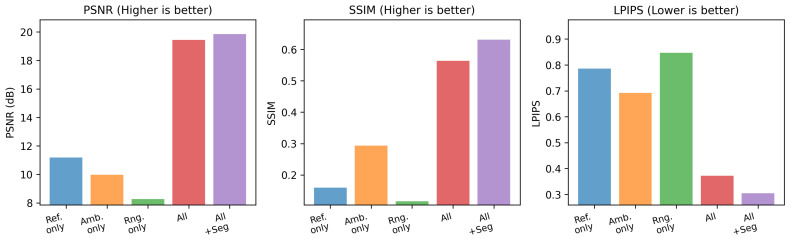
Bar plots comparing PSNR, SSIM, and LPIPS across five LiDAR-input configurations. “Range-only” performs significantly worse both in pixel-based metrics and in perceptual quality. In contrast, “all+seg” attains the highest PSNR (19.85 dB), best SSIM (0.63), and the lowest LPIPS (0.31), underscoring the value of fusing multiple LiDAR modalities and semantic constraints.

**Figure 12:**
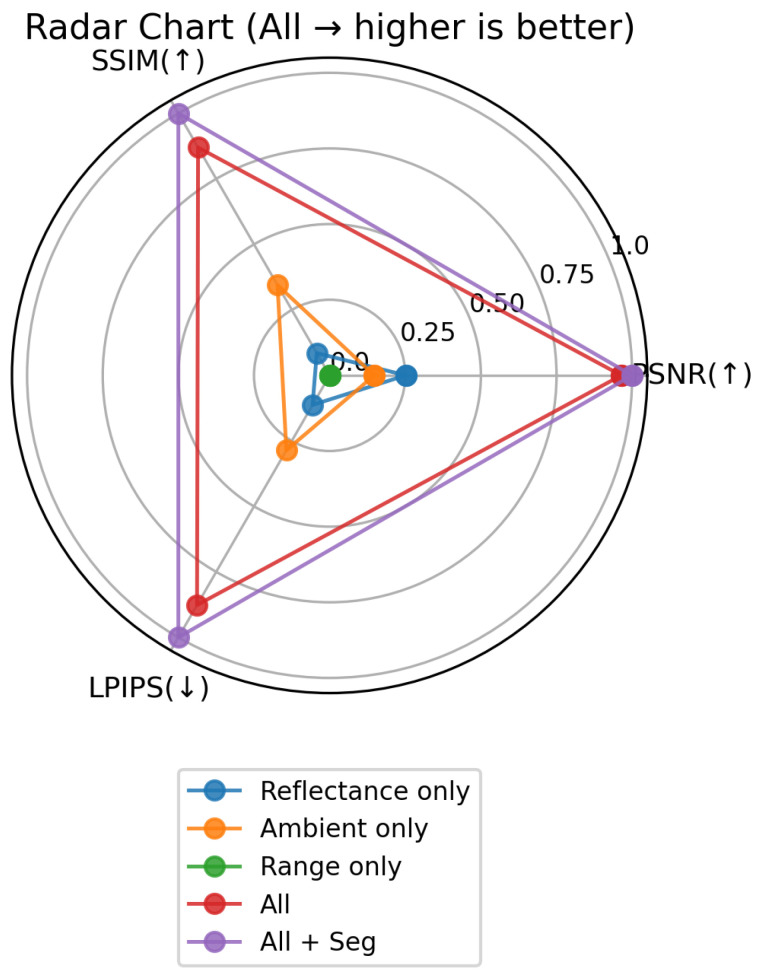
Radar chart showing normalized PSNR, SSIM, and inverted LPIPS for each model variant. Larger radii consistently indicate better performance. The up-arrow (↑) signifies that higher values are better, and the down-arrow (↓) signifies that lower values are better.

We evaluate five experimental variants: reflectance only, ambient only, range only, all, and all+seg (the same three channels with an additional segmentation-based loss). When a channel is unused, it is replaced by a zero-valued image to mask out that modality. Each model is trained and tested on the DurLAR dataset (Section 4.4) with a 5% random temporal split and neighbor-discarding strategy. Table 1 summarizes PSNR, SSIM, and LPIPS on the held-out test frames. Figure 11 displays these metrics via bar plots, and Figure 12 employs a radar chart where LPIPS is inverted so that a larger radial value consistently signifies superior performance.

Examining the single-modality baselines reveals clear limitations: range-only achieves the lowest PSNR (8.2648 dB) and SSIM (0.1160) while yielding the highest LPIPS (0.8470). Likewise, reflectance-only and ambient-only perform better than range-only but still lag far behind the multi-channel versions. By contrast, combining reflectance, ambient, and range all substantially boosts reconstruction fidelity (PSNR = 19.4384 dB, SSIM = 0.5639, LPIPS = 0.3721). Further incorporating segmentation-based guidance all+seg improves PSNR and SSIM modestly but reduces LPIPS from 0.3721 to 0.3052—an indicator of more perceptually convincing outputs.

Overall, these results underscore the importance of fusing multiple LiDAR modalities for camera-image reconstruction. The additional segmentation-based loss, while not drastically altering pixel-level metrics, provides a notable reduction in LPIPS, aligning more closely with human perception.

To assess reproducibility, we repeated the full training pipeline using different random seeds on a smaller subset. Standard deviations of all metrics remained under 0.15 dB for PSNR and under 0.02 for SSIM, suggesting stable convergence. Although the absolute numbers vary modestly across seeds, the relative ordering among model variants persists.

Although the software-based redundancy strategy can reduce the monetary costs associated with hardware redundancy (i.e., adding additional perception sensors), it introduces additional computational overhead. The cost-effectiveness of adding sensors versus increasing computational resources largely depends on the pace of advancements in both sensor and GPU technologies. This paper proposes a software-based redundancy approach as an alternative to, or complement to, hardware-based strategies, offering an additional means to enhance system redundancy. Furthermore, since the software-based redundancy operates on GPUs already integrated into Level 4 and higher ADS platforms, no extra hardware is required to implement the redundancy. This approach also helps address the aesthetic challenges of hardware-based redundancy by eliminating the need to mount additional sensors on the vehicle physically.

To assess the practical viability of our approach, we benchmarked the computational performance of our final model. The inference speed was measured on an NVIDIA RTX 2080Ti GPU with compute power of 13.45 TFLOPS. We specifically evaluated the network’s forward pass latency, a standard measure of a model’s core efficiency, which excludes data pre-processing tasks (e.g., LiDAR point cloud projection) that can often be parallelized on the CPU. Our generator achieves an average inference time of 10.81 ms per frame. This corresponds to a throughput of 92.5 FPS. This high throughput highlights the potential of our model for integration into real-time autonomous driving pipelines in future Level 4 and higher autonomous vehicles equipped with computational power exceeding 2000 TFLOPS [43].

### 5.2. Qualitative Evaluation

**Figure 13:**
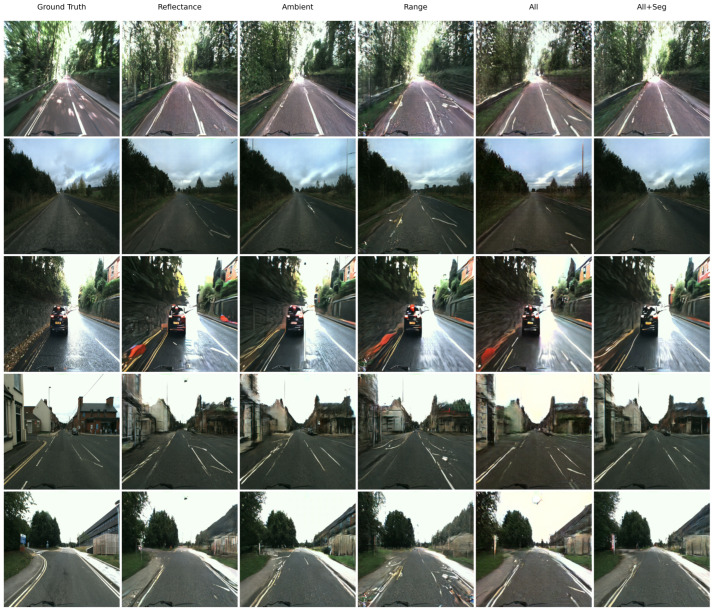
Representative results on unseen DurLAR frames. Each column displays the ground-truth camera image alongside reconstructions from five expirements setups: conditioned on reflectance only, ambient only, range only, all three LiDAR modalities (all), and all three modalities with semantic segmentation loss (all+seg). Results show progressive improvement: single-modality inputs have limitations, while their fusion (all) enhances details (e.g., façades, road markings). The all+seg variant (right-most column) yields the most significant gains; semantic guidance improves clarity of fine structures, suppresses artifacts, and produces the most faithful overall reconstructions, particularly for architectural elements and vehicles.

Beyond quantitative metrics, qualitative analysis provides crucial visual insights into LiGenCam’s performance across different input configurations. Figure 13 showcases representative reconstructions from unseen DurLAR test frames, encompassing diverse scenarios such as road scenes (rows 1–2), a vehicle in an urban context (row 3), and detailed architectural elements (rows 4–5). This visual examination allows for a nuanced assessment of how different LiDAR modalities and their fusion, with and without semantic guidance, impact the final image fidelity.

Reconstructions from single LiDAR modalities highlight their individual limitations and distinct contributions. Relying solely on range data, which primarily conveys geometric depth, results in the poorest image quality, marked by severe blurring, significant loss of structural detail (e.g., the building in row 5 is misinterpreted as foliage, and rooftops in row 4 are erroneously rendered with vegetation), and inaccurate rendering (e.g., noisy road markings in row 1). Despite these flaws, the model exhibits a rudimentary ability to associate broad color regions with spatial locations, suggesting a coarse learned mapping between depth and general color categories, though this is insufficient for detailed reconstruction.

Utilizing either reflectance or ambient LiDAR data as single-channel inputs offers a substantial improvement. These modalities provide intensity information related to material surface properties (reflectance) or near-infrared illumination (ambient), enabling the capture of finer object details and more coherent scene structures, leading to visually acceptable reconstructions. However, their distinct sensing principles lead to different strengths and artifacts. For instance, in the architectural scene of row 4, reflectance inputs also incorrectly introduce vegetation on rooftops, similar to range, albeit with slightly better structural definition. Ambient data, in the same scene (row 4), reconstructs the building structures more reasonably but may not achieve optimal color fidelity. Elsewhere, the ambient-based reconstruction in row 2, while accurately rendering the road surface, erroneously introduces a utility pole. Conversely, reflectance-based outputs occasionally struggle with consistent coloration (e.g., the reddish tint on the line in row 3) or exhibit blurriness in complex textures like the building in row 5. Both modalities show variability in precisely rendering fine details like road markings (row 1), indicating that single-channel intensity information alone faces challenges in resolving complex patterns or avoiding minor hallucinatory elements.

Fusing all three LiDAR modalities (reflectance, ambient, and range) via early concatenation allows LiGenCam to leverage their complementary strengths, yielding a notable enhancement in overall reconstruction quality. This approach mitigates some weaknesses of single-modality inputs, as structural guidance from range data, augmented by textural and material cues from reflectance and ambient, generally produces sharper object delineations and more consistent scene geometry. Nevertheless, as seen with road markings in row 1, this direct fusion can still produce some visual inconsistencies. For the building scene in row 4, while generally improved over single modalities, the all configuration may still exhibit minor color inaccuracies or less crisp details compared to the semantically guided version, suggesting that further high-level guidance is crucial for optimal semantic and pixel-level accuracy.

The introduction of a semantic segmentation loss (all+seg) provides this critical high-level contextual understanding, guiding the generator to produce images that are both visually compelling and semantically sound, representing the best performance among all configurations. This semantic guidance encourages the network to generate images whose segmented content closely aligns with that of the ground-truth. The impact is evident across various critical elements: road markings are rendered with superior precision, including correct delineation of features like dashed line spacing (row 1); vehicles (row 3) exhibit sharper contours and more accurate coloration. Crucially, for architectural scenes (rows 4 and 5), the all+seg variant demonstrates superior capabilities. In row 4, it most accurately reconstructs the building, correctly rendering rooftops without the erroneous vegetation seen in range and reflectance outputs, and achieves the best color rendition compared to ambient and all. Similarly, structures in row 5 are restored with significantly improved structural integrity and material appearance. By enforcing consistency at the semantic class level, this approach not only helps in accurately distinguishing and delineating objects but also implicitly improves intra-class textural details and inter-class boundaries, effectively suppressing artifacts and enhancing perceptual realism, an observation that aligns with the quantitative LPIPS improvements.

In summary, the qualitative results presented in Figure 13 visually corroborate the quantitative findings, demonstrating a clear hierarchy of performance. Progressively enriching the input LiDAR data and, critically, incorporating semantic consistency constraints, systematically enhances the fidelity and perceptual quality of the reconstructed camera images. The all+seg configuration of LiGenCam consistently yields the most accurate and perceptually convincing results, underscoring the value of combining multimodal sensor fusion with semantic-aware training objectives for robust image reconstruction in autonomous driving applications.

## 6. Limitations and Future Works

Although LiGenCam demonstrates promising performance across multiple modalities, it is not without limitations. One notable restriction is the fixed input and output resolution of 512×512. This choice strikes a balance between computational feasibility and reconstruction quality, but real-world ADS often demands higher resolutions for fine-grained scene understanding. Future research may explore higher-resolution training, potentially with larger model architectures and more advanced hardware to capture richer details in challenging urban environments.

Another limitation stems from the reliance on ambient modalities, which are not universally supported by all LiDAR sensors. Moreover, the projected LiDAR images remain a simplification. A more direct, end-to-end approach that maps raw 3D point clouds to camera images could better exploit the full spatial distribution of LiDAR points, although it would require additional computational resources.

While incorporating a segmentation-based KL divergence shows clear benefits, the method treats all semantic classes equally. In safety-critical domains such as ADS, certain classes (e.g., “car,” “pedestrian,” or “bicycle”) warrant greater attention and precision. Future work could explore class-weighted segmentation losses or more sophisticated weighting strategies that prioritize objects essential for driving tasks. A finer class-specific weighting factor, for instance, might further reduce errors around vehicles or other relevant traffic participants.

Recent advances in diffusion models [44] also indicate their potential to surpass GAN-based methods in image synthesis quality and training stability. Switching to a diffusion-based LiGenCam variant may help eliminate artifacts and reduce dependence on adversarial balancing. Finally, our current system does not enforce temporal consistency for sequential frames, which can cause flickering or inconsistent object boundaries. Integrating a temporal module, such as a Transformer-based network [45], could provide smoother, more coherent video-like outputs for real-time ADS applications.

## 7. Conclusions

In this paper, we presented LiGenCam, a novel method for reconstructing camera images from multimodal LiDAR data. The ability to generate reliable camera views under circumstances where optical cameras may fail can significantly enhance sensor redundancy for ADS. Our approach leverages three LiDAR-based modalities—reflectance, ambient, and range—together with a semantic segmentation loss, allowing LiGenCam to achieve both geometric accuracy and semantic consistency in its reconstructions.

Experiments on the DurLAR dataset demonstrate that incorporating all three LiDAR modalities yields more perceptually convincing results than single-modality baselines, as supported by improved LPIPS scores. Furthermore, imposing segmentation-based constraints (all+seg) consistently refines the reconstruction of critical objects, demonstrating notable improvements in the structural integrity and visual fidelity of complex elements such as vehicles and architectural features like buildings, thus validating the importance of semantic alignment for safety-critical domains. These findings underscore the promise of fusing multiple LiDAR-derived inputs to restore camera perspectives in real-world driving environments.

Despite the encouraging performance, several limitations remain. The resolution of 512×512 may be insufficient for complex urban settings requiring finer visual details. Additionally, certain classes like “car” and “pedestrian” could benefit from targeted weighting, enhancing the accuracy of object-critical regions. Future work may thus explore higher-resolution training, class-specific segmentation penalties, or alternate generative frameworks such as diffusion models to further elevate reconstruction fidelity. By addressing these challenges, LiGenCam could evolve into a robust failsafe for ADS camera subsystems, safeguarding navigational decisions against sensor malfunctions and ultimately contributing to a more resilient autonomous driving experience.

## Figures and Tables

**Table 1:** Quantitative results on the DurLAR test set. Higher PSNR/SSIM is better, lower LPIPS is better.

Method	PSNR (dB) ↑	SSIM ↑	LPIPS ↓
Reflectance only	11.1819	0.1598	0.7859
Ambient only	9.9761	0.2934	0.6921
Range only	8.2648	0.1160	0.8470
All (no Seg)	19.4384	0.5639	0.3721
All + Seg	**19.8535**	**0.6310**	**0.3052**

The best results are highlighted in bold. The up-arrow (↑) indicates that higher values are better, and the down-arrow (↓) indicates that lower values are better.

## Data Availability

The dataset used in this study is publicly available at https://github.com/l1997i/DurLAR (accessed on 6 July 2025).
